# Subtle changes in striatal muscarinic M1 and M4 receptor expression in the DYT1 knock-in mouse model of dystonia

**DOI:** 10.1371/journal.pone.0226080

**Published:** 2019-12-05

**Authors:** Franziska Richter, Laura Klein, Christin Helmschrodt, Angelika Richter

**Affiliations:** 1 Institute of Pharmacology, Pharmacy and Toxicology, Faculty of Veterinary Medicine, University of Leipzig, Leipzig, Germany; 2 Department of Pharmacology, Toxicology, and Pharmacy, University of Veterinary Medicine Hannover, Hannover, Germany; University of Florida, UNITED STATES

## Abstract

In early-onset generalized torsion dystonia, caused by a GAG deletion in TOR1A (DYT1), enhanced striatal cholinergic activity has been suggested to be critically involved. Previous studies have shown increased acetylcholine levels in the striatum of DYT1 knock-in (KI) mice. Ex vivo data indicated that muscarinic receptor antagonists normalize the activity of striatal cholinergic interneurons. Currently receptor subtype specific antagonists are developed for therapy, however, it is yet unknown whether the levels of targeted receptors are unaltered. In the present study, we firstly examined the expression of M1 and M4 receptors in DYT1 KI mice in comparison to wildtype mice. While no changes in mRNA were found in the motor cortex, the expression of M1 was higher in the striatum of DYT1 KI. However, M1 protein did not differ in striatum and cortex between the animal groups as shown by immunohistochemistry and western blot. M4 receptor protein, unaltered in the cortex, was slightly lower in lateral subparts of the striatum, but unchanged in somata of cholinergic interneurons and substance P immunoreactive projection neurons. Functional alterations of the cholinergic system and of aberrant striatal plasticity, demonstrated by previous studies, seem not to be related to overt changes in M1 and M4 expression. This critically informs the ongoing development of respective antagonists for therapy of dystonia.

## Introduction

Various types of generalized dystonia, characterized by sustained or intermittent involuntary movements, are regarded as a network disorder that involves corticostriatal dysfunctions and abnormal basal ganglia outflow [1, 2]. The pathophysiology of early-onset generalized torsion dystonia, caused by a GAG deletion in TOR1A (DYT1) with low penetrance, is not known. However, in line with clinically used muscarinic 1 (M1) receptor preferring antagonists in human DYT1 dystonia, a series of ex vivo experiments in DYT1 animal models indicated a paradoxical excitation of striatal cholinergic interneurons (ChI) to normally inhibitory dopamine D2 receptor activation [3]. In DYT1 knock-in (KI) mice, which do not develop dystonic symptoms like other viable DYT1 models [4], extracellular acetylcholine was found to be increased in the striatum and blocking of acetylcholine receptors normalized D2 receptor mediated effects on striatal ChI [5]. In addition to these interesting findings, our data on in vivo optogenetic stimulations of striatal ChI supported an endophenotype of dysregulated cholinergic activity, although depolarizing of these interneurons was not sufficient to induce overt dystonia in DYT1 KI mice [6].

The expected response to a hypercholinergic tone is receptor internalization, generally followed by overall downregulation of receptor mRNA and protein expression [7]. Whether abnormal expression of muscarinergic (M) receptors is involved in the hypercholinergic state in DYT1 KI mice has not been examined yet. In order to extend the knowledge on striatal cholinergic dysfunctions in DYT1 dystonia, we examined the expression of cortical and striatal M1 and M4 receptors in DYT1 KI mice in the present study. Cholinergic activation of these receptors plays an important role in motor control [8, 9]. M1 receptors are coupled to Gq/11 (as M3 and M5) and are localized on striatal projection neurons (SPN). M4 receptors (and M2), coupled to Gi/o proteins, are expressed postsynaptically on striatonigral SPN, on glutamatergic terminals and presynaptically on ChI, where they mediate a negative feedback control on acetylcholine release [3, 10].

## Materials and methods

### Animals

Animal care and experiments were in accordance with the German Animal Welfare Agency and the European guidelines (Directive 2010/63/EU) and approved by the local ethics committee and authority (Landesdirektion Sachsen TVV20/13). Male six-month-old heterozygous DYT1 (ΔGAG) knock-in mice (DYT1 KI) [11] and wildtype (littermates or from same line) were used (C57Bl/6J background), total of n = 12 per genotype. They were bred and housed in the institute’s facility in groups up to 6 littermates. Genotypes were assessed by polymerase chain reaction (PCR) amplification analysis of DNA extracted from ear tissue using PuReTaq Ready-To-Go Beads (GE Healthcare) as described previously [6].

Mice were bred and group-housed in the facility of the institute (Leipzig) on a 12h light/12h dark cycle in makrolon cages (Type III, not ventilated and open to environment) at 24°C ± 2°C with relative humidity of about 60%. Food (Altromin standard diet) and water were available ad libitum and material for nest building was provided. The number of mice used in each experiment was calculated in a priori power analysis (GPower 3.1) and is provided together with the results in the figure legends (n = 6 per group).

### Quantitative real-time PCR (qPCR)

Quantification of mRNA was done as described previously (e.g. [12, 13]). Briefly, mice (n = 6/genotype) were deeply anesthetized with intraperitoneal injection of 100 mg/kg pentobarbital and perfused transcardially with 0.1 M NaCl. Brains were removed, embedded in tissue tek and snap-frozen in -20°C 2-methylbutane. Striatum and cortex were dissected from frozen tissue and stored at -80°C. Total RNA was isolated (RNeasy Plus Mini Kit, Qiagen, CA, USA) and a defined amount used for first-strand cDNA preparation (Takara Clontech 5X Primescript RT Master Mix, Takara Bio USA, Inc.) followed by real time qPCR (TaqMan®Universal PCR Master Mix, Thermo Fisher Scientific, Waltham, USA) in a PikoReal 96 Real-Time PCR system and software (Thermo Fisher Scientific, Waltham, USA). The following TaqMan Gene Expression Assays were used: Chrm1 (Mm00432509), Chrm4 (Mm00432514) and for house-keeping Gapdh (Mm99999915, house-keeping Gen), Hprt (Mm01545399, house-keeping Gen), Eif4a2 (Mm00834357, house-keeping Gen), Atp5b (Mm00443967, house-keeping Gen). GeNorm was used to calculate expression stability and normalization factors [12].

### Western blotting

Western blot quantification of M1 receptor protein was performed following previously published protocols [12] on the striatum and motor cortex using tissue dissected from frozen brains of the same mice as used for mRNA analysis (n = 6/genotype). In brief, protein was extracted in 20 mM Tris∙HCl, 150 mM NaCl, pH 7.4 and protein concentration was determined by BCA protein assay (Thermo Fisher Scientific, Germany). Proteins were fractionated via SDS-PAGE on 4–15% SDS-polyacrylamid gel (Mini-PROTEAN TGX, Bio-Rad) and then transferred to PVDF membrane with a semi-dry transfer unit (Bio-Rad). Membrane was blocked (StartingBlock T20, Thermo Scientific; #37539, 1h) incubated with anti M1 receptor antibody (rabbit polyclonal AB5164, Millipore) at 1:1,000 dilution or mouse anti-actin (MAB1501; dilution 1:2,000; Merck Chemicals GmbH) in blocking buffer for 1 h at room temperature. Then, the membranes were washed with PBST (0.2% Tween-20 in 0.1 M phosphate-buffered saline) and incubated with anti-rabbit and anti-mouse secondary, horseradish peroxidase-conjugated antibody (1:5,000; Jackson Immunoresearch, Suffolk, UK) for 1 h at room temperature, washed with PBST, and developed with a chemiluminescent substrate (SuperSignal West Pico; Thermo Scientific, Rockford, IL). Blots were visualized (FUSION Advance, Peqlab, Germany) and resulting bands were quantified using ImageJ analysis software (NIH). The optical density values (OD) were normalized to loading control actin.

### Immunohistochemistry (IHC)

Tissue was processed for IHC based on previously published protocols [6, 14, 15]. Mice (n = 6/genotype) were deeply anesthetized with intraperitoneal injection of 100 mg/kg pentobarbital and perfused transcardially with 0.1 M phosphate buffered saline (PBS) followed by 4% paraformaldehyde in 0.1 M PBS (Pfa). Brains were removed and postfixed overnight by 4°C in Pfa. Brains were equilibrated for 3 days in an increasing sucrose dilution from 10 to 30% in 0.1 M PBS, frozen and stored at -80°C. The brains were cut in 40 μm sections on a cyrostat (Hypax C 50, Zeiss, Germany) and stored at -20°C in cryoprotectant solution (500 ml: 250 ml 0.1 M PBS, 250 ml glycerol, 0.33 g MgCl_2_, 42.8 g sucrose). Medial striatal sections were used for all labeling. Free-floating sections were washed in 50 mM tris buffer solution (TBS, pH 7.6), followed by a blocking solution with 0.5% Triton X-100 (Tx100) and 10% normal donkey serum (NDS) in TBS for 1 h. Sections were incubated with primary antibody (all from Merck Millipore, Darmstadt, Germany): anti M1 receptor (rabbit polyclonal 1:100, AB5164) or anti M4 receptor (mouse monoclonal 1:500, MAB1576) in 2% NDS in TBS 24–48 h at 4°C. M1 and M4 receptor specific antibodies used in this study were extensively characterized for high specificity and absence of cross reaction with other than the specified receptor in the muscarinic family [16, 17], and protocols were previously established [15]. As negative control the primary antibody was omitted. For co-labeling choline acetyltransferase (ChAT, goat polyclonal 1:500, AB144P) or anti substance P (SP, rabbit polyclonal 1:500, AB1566) were added. Both antibodies were previously tested and specificity of the protocol established [6]. Thereafter, sections were washed and incubated with AlexaFluor 488 or 594 conjugated secondary antibodies (1:500–800, donkey anti-rabbit, anti-mouse or anti-goat, Jackson Immuno Research, Suffolk, UK) in 2% NDS and 0.5% Tx100 in TBS for 1 h. After a final wash, sections were mounted on glass slides and coverslipped with VectaShield Mounting Medium (H-1000, Vector Laboratories, CA, USA). Slides were stored at 4°C and protected from light.

For examination of expression and distribution of M1 receptors or M4 receptors, a Zeiss Axioskop microscope and the Retiga 2000R CLR-12 digital camera was used. Both hemispheres of two medial striatal sections per animal were divided into 4 subregions (dorsomedial, dorsolateral, ventromedial and ventrolateral) as done previously (e.g. [18]). From the center of each striatal subregion and from layer V of the motor cortex an image was acquired using the 40x objective. Image acquisition parameters were kept constant between animals and regions. Analysis of the images was done with the software Image J (NIH, USA), measuring the mean pixel fluorescence intensity of each region. Values between left and right hemisphere and between the two sections did not differ and were averaged for further analysis. All analyses were undertaken by an investigator who was unaware of the genotype of the animals. Co-localization of M4 receptors and ChAT or SP positive signal was verified using a confocal microscope (Olympus Fluoview FV 1200, Software Olympus Fluoview 4.1, Olympus, Germany) for image acquisition across the striatum at 40x. Image acquisition parameters were kept constant during quantification. Fluorescence intensity of M4 receptors, ChAT or SP was measured in ChAT or SP positive neurons using ImageJ as described previously [6]. Specifically, in images from striata of both hemispheres, each ChAT- or SP-positive neuron with a nucleus was identified and outlined, excluding the nucleus from the area to be measured. M4 immunofluorescence intensity measurements were collected from the outlined area for individual CHAT- or SP-positive neurons in composite images.

### Statistics

All data were acquired and analyzed by an investigator who was blind to group conditions. Data was tested for normality and homogeneity of variance prior to executing the respective statistical test (SigmaPlot 12.0). mRNA and protein expression were analyzed with a parametric mixed design analysis of variance (ANOVA) (genotype x region) with region as repeated measure followed by multiple comparisons correcting post-hoc test (Holm-Sidak method). Student´s t-test was used for comparing IHC results of the cortex and co-labeling data of the striatum. If data were normalized then statistics were performed on raw data. Significance was assigned at p<0.05.

### Results and discussion

In view of therapeutic benefit of M1-preferring receptor antagonists in some patients with isolated, generalized dystonia [19], strong evidence for striatal cholinergic dysregulation in DYT1 models such as DYT1 knock-in mice [3, 6], and the hypothesis that M4 receptors could be an interesting target for the treatment of dystonia [15, 20], we determined the expression of M1 and M4 receptors in the striatum and motor cortex in the DYT1 KI model in the present study. As recently found in other animal models of generalized dystonia, i.e. in *dt*^*sz*^ mutant hamsters with paroxysmal dystonia [15] and in a knock-in mouse model of L-DOPA-responsive dystonia [21], the present data only show subtle changes in striatal M1 and M4 receptor expression in DYT1 KI mice.

**Fig 1:**
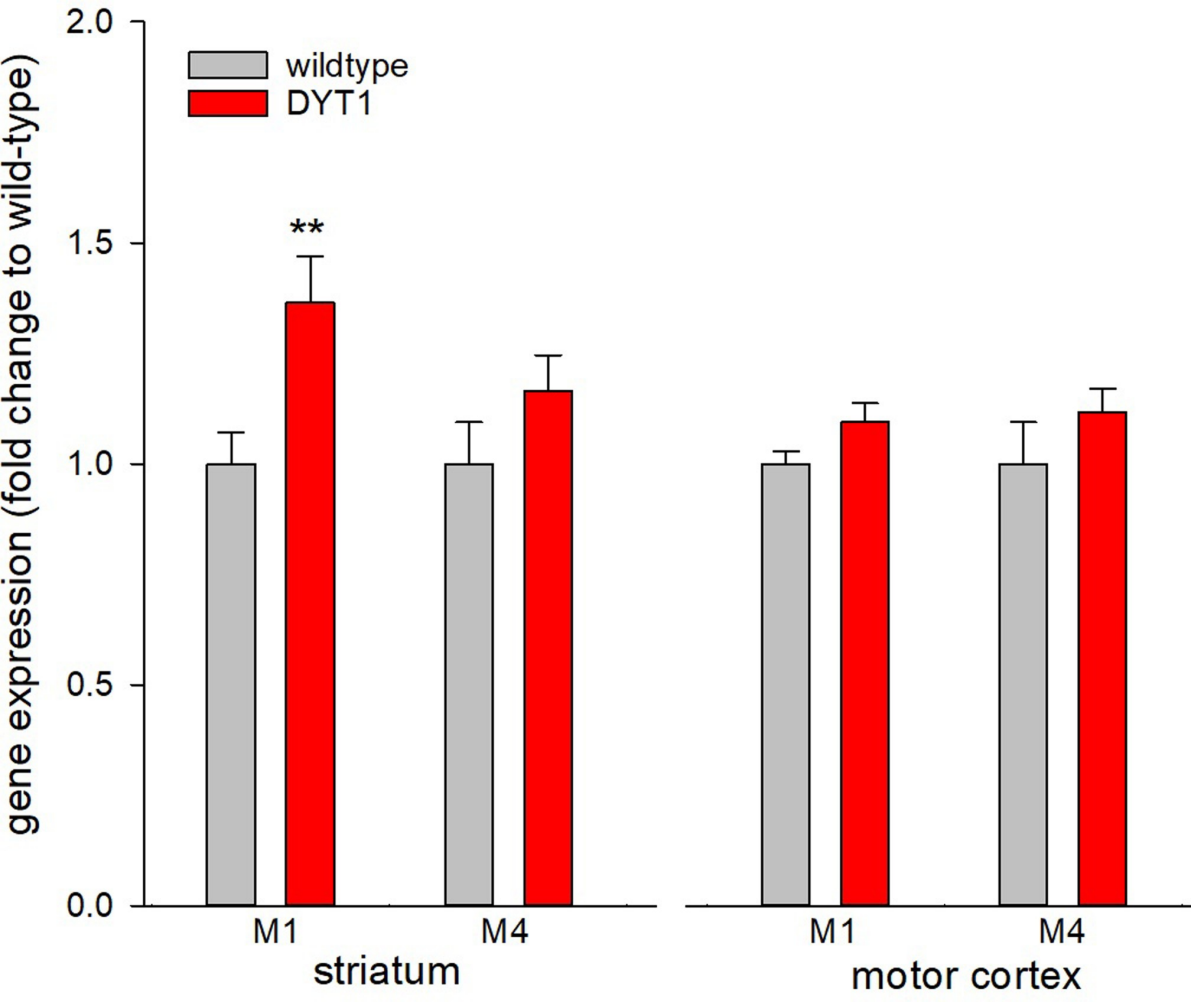
Receptor mRNA expression. Relative quantification of M1 and M4 receptor mRNA expression in the striatum and motor cortex of DYT1 KI (DYT1, red bars) and wildtype mice (WT, grey bars). Values (mean + S.E.M.) are shown normalized to means of control groups to visualize fold change; Holm Sidak, Two Way RM ANOVA, **p<0.01 to wildtype, statistics were performed on raw data, n = 6 each.

Relative expression quantification of M1 receptor mRNA revealed an increase in striatum but not cortex of DYT1- KI versus wildtype littermates (ANOVA genotype F(1/9) = 17, p<0.001, region F(1/9) = 20, p<0.002; Fig 1).

**Fig 2:**
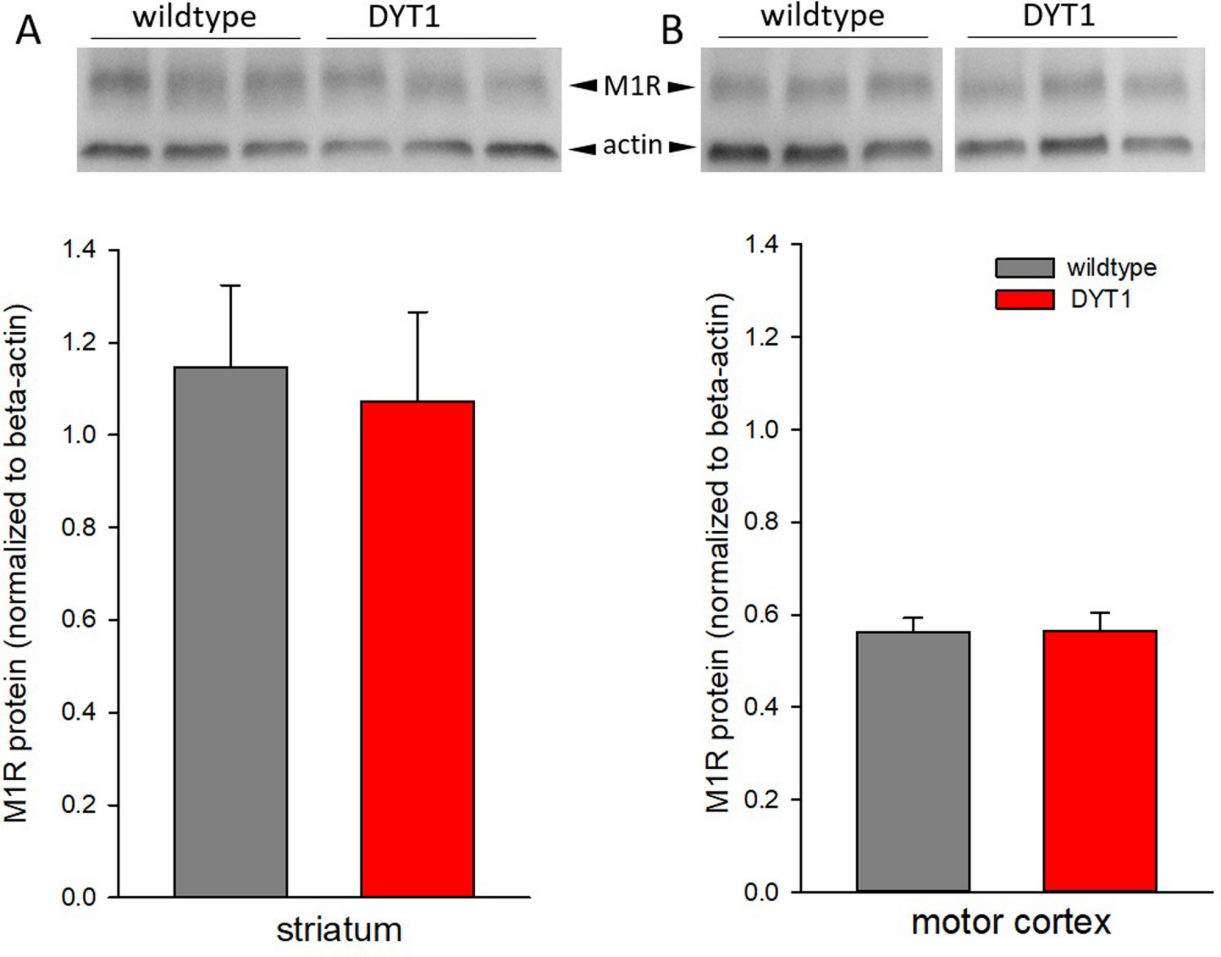
M1 receptor protein expression. Western blot of M1 receptors in the (A) striatum and (B) motor cortex of DYT1 KI and wildtype mice. Representative blots with bands for M1 receptor ~ 51 kDa and actin ~ 40 kDa. No differences in expression between genotypes (mean + S.E.M.), M1 receptor expression was normalized to actin for quantification, n = 6 each.

In order to confirm M1 receptor mRNA increase on protein level western blotting was performed (Fig 2). The protocol resulted in a single band for M1R at 51 kDa representing the monomeric protein. There were no differences in M1 receptor protein expression either in striatum or in cortex.

**Fig 3:**
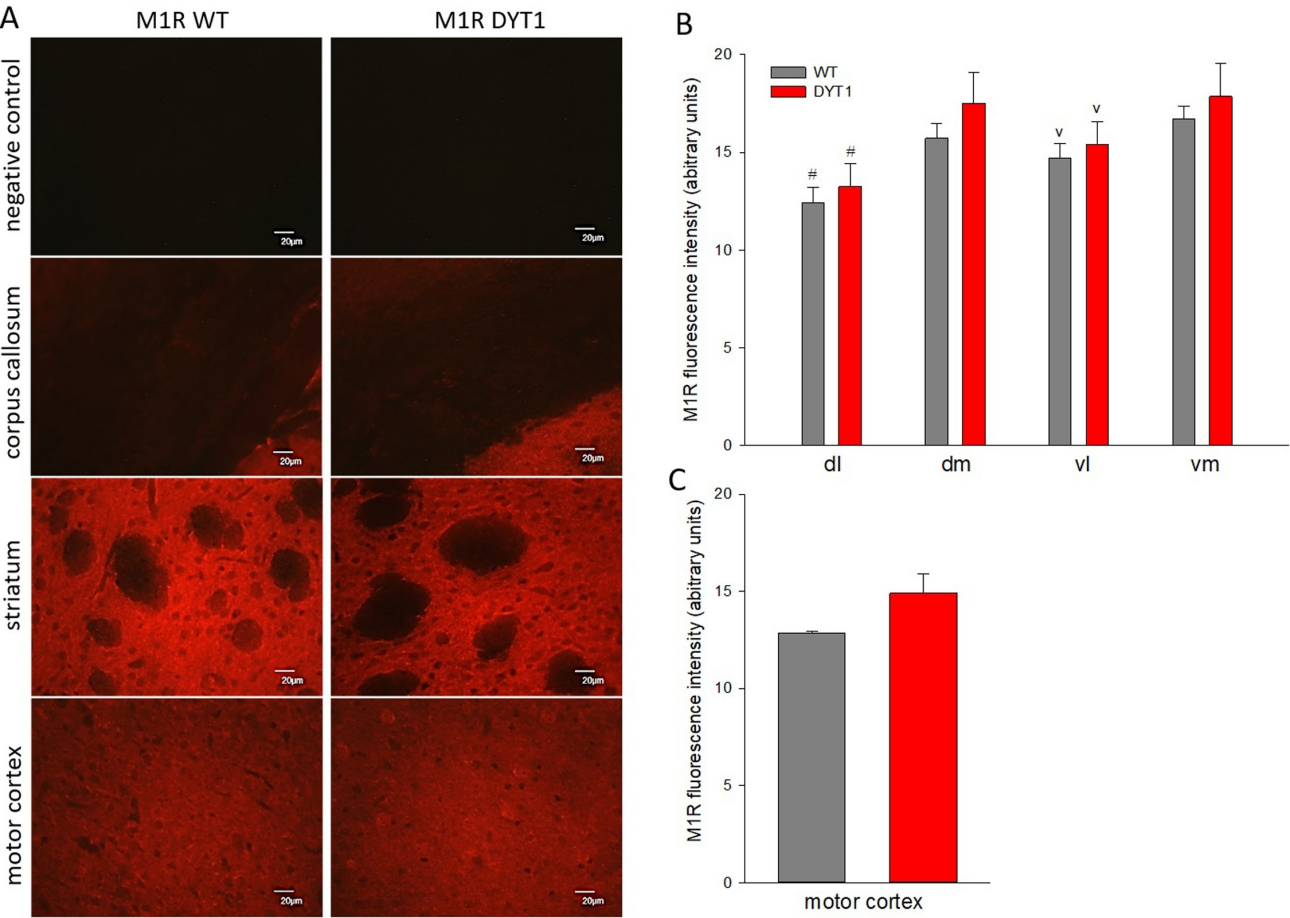
M1 receptor immunohistochemistry. (A) Representative images of M1 receptor (red) in striatum and motor cortex of DYT1 KI (DYT1) and wildtype (WT) mice, scale bar is 20 μm; negative control without primary antibody and corpus callosum show no specific staining as expected; quantification of M1 fluorescence intensity in the (B) striatum divided in subregions, dl: dorsolateral, dm: dorsomedial, vl: ventrolateral, vm: ventromedial or (C) motor cortex; Two Way RM ANOVA, # p<0.05 to all other regions, v p<0.05 to dm and vm, means + S.E.M., n = 6 each.

Subregion specific receptor protein expression and distribution were further quantified using the same M1R specific antibody in established immunohistochemistry protocols. M1 receptor expression was high in the striatum, comparably lower in the cortex and absent in the corpus callosum and in negative controls with primary antibody omitted (Fig 3), as expected and previously characterized [15]. Contrary to mRNA expression but confirming western blot data, there were no significant genotype differences in M1 receptor protein expression in striatal subregions or cortex (Fig 3).

Thus, the protein level of M1 receptors was unaltered in the striatum and motor cortex, although striatal M1 receptor mRNA was increased in the DYT1 mutant in comparison to wildtype mice. In DYT1 mutant mice, microdialysis revealed increased striatal acetylcholine levels [5]. In many cells, exposure to muscarinic agonists induces rapid desensitization and internalization of the cell surface muscarinic receptors followed by a loss of total number of cellular receptors in the following hours. This receptor down-regulation involves protein and mRNA degradation and can only be overcome by de novo receptor synthesis [7]. Furthermore, long lasting increases of extracellular acetylcholine levels induced by acetylcholineesterase (AChE) inhibition lead to reductions of muscarinic receptors in the striatum [22, 23]. Based on these evidences we expected downregulation of muscarinic receptors in hypercholinergic DYT1 KI mice. The instead observed increase in M1 mRNA originating from presumably dysregulated medium spiny neurons, supports a more complex regulation of cholinergic transmission, likely involving dopamine receptor signaling and converging molecular pathways [24]. It is widely appreciated that transcript and protein levels often do not correlate; however, in summary our data supports that changes in M1R expression are unlikely to underlie the aberrant corticostriatal plasticity with higher long-term potentiation in DYT1 mice normalized by M1-preferring antagonists [25].

**Fig 4:**
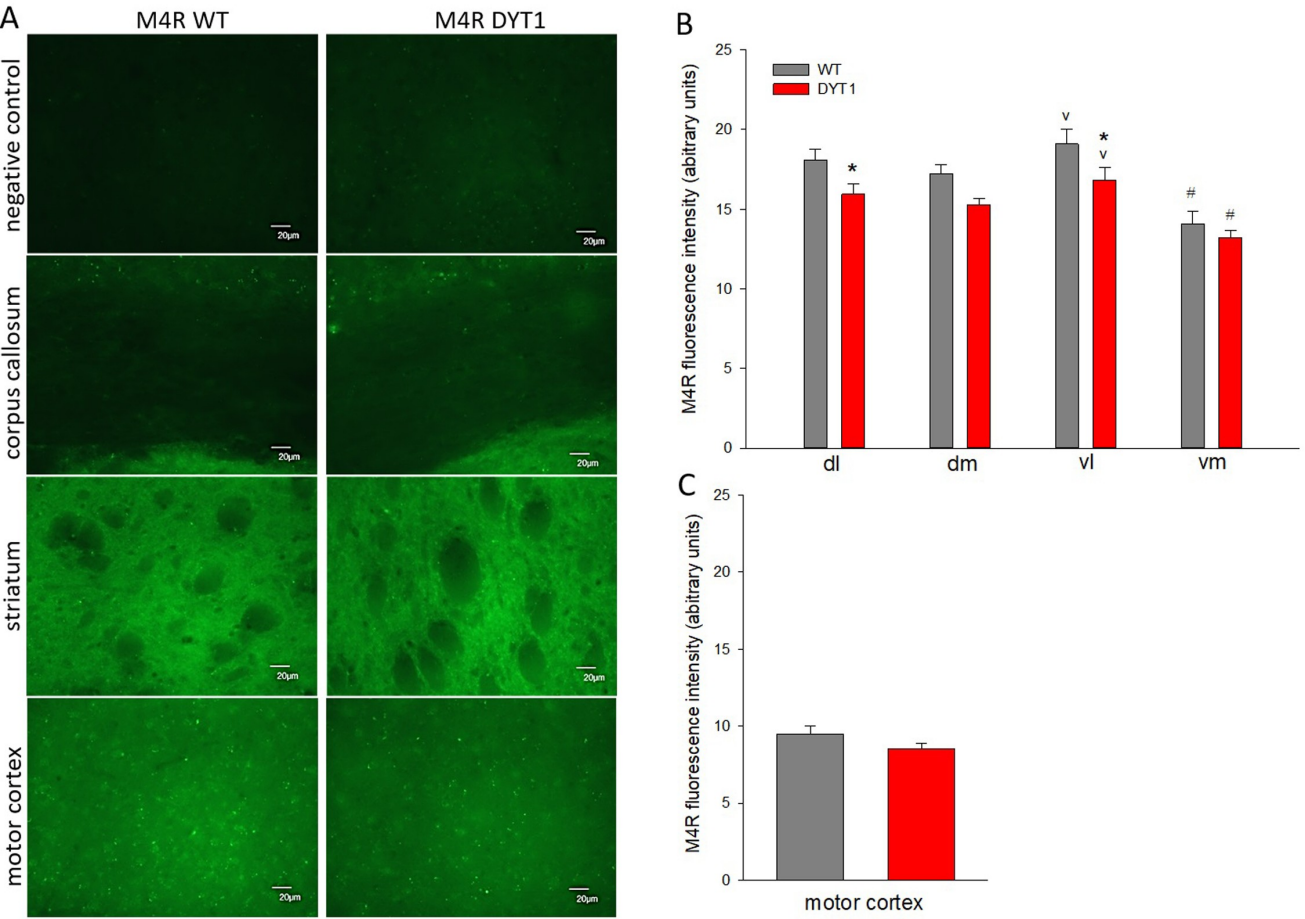
M4 receptor immunohistochemistry. (A) Representative images of M4 receptor (green) in striatum and motor cortex of DYT1 KI (DYT1) and wildtype (WT) mice, scale bar is 20 μm; negative control without primary antibody and corpus callosum show no specific staining as expected; quantification of M4 receptor fluorescence intensity in the (B) striatum divided in subregions, dl: dorsolateral, dm: dorsomedial, vl: ventrolateral, vm: ventromedial or (C) motor cortex; Two Way RM ANOVA, #p<0.05 to all other regions, vp<0.05 to dm, *p<0.05 to wildtype, means + S.E.M., n = 6 each.

Other counteracting mechanisms to a hyperactive cholinergic state consist in an up-regulation of the acetylcholine degrading enzyme AChE or of M4 autoreceptors, localized on ChI. Our recent in vivo optogenetic studies revealed higher AChE fluorescence intensity after stimulation of ChI in DYT1 KI mice, but there were no basal changes compared with wildtype mice [6]. In this previous study we stimulated ChI via optogenetics and found a prolonged c-Fos activation of these neurons in DYT1 KI mice [6]. M4 receptors on ChI may be involved in this hypercholinergic state, as they have been described to facilitate ACh release instead of the expected physiological autoreceptor role of transmitter release inhibition [3]. Therefore, we studied whether changes in M4R expression levels may be involved. As shown in Fig 1, there were no differences in M4R mRNA quantity in the striatum. This is in line with unaltered levels of M4R in western blots from another line of DYT1 KI mice [26]. Because of the above described ChI specific phenotype of DYT1 KI mice one could expect subregional and neuronal subtype specific alterations of M4R expression in the striatum. Since western blotting of the entire striatum cannot capture these specific changes, we quantified M4 receptor fluorescence intensity in different striatal subregions and in individual ChI (ChAT-positive) or direct SPN (SP-positive) in the present study. The M4R receptor specific antibody and the protocol have been used previously [15] and specificity was as expected with strong staining in striatum, weak staining in the cortex and absence of specific staining in corpus callosum and negative control with primary antibody omitted (Fig 4). A small decrease in fluorescence intensity only in the dorsolateral and ventrolateral subregion of the striatum was observed in DYT1 KI mice (ANOVA genotype F(1/30) = 4.7, p = 0.055, region F(1/30) = 42, p<0.001, Fig 4).

**Fig 5:**
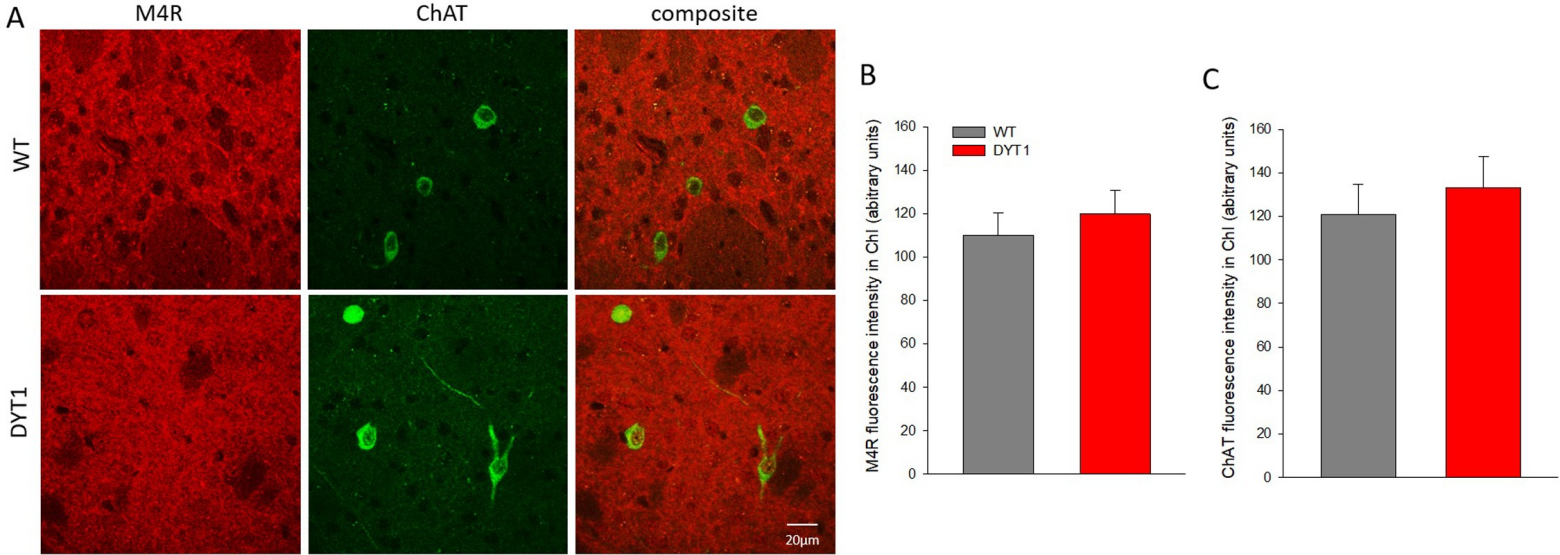
M4 receptor in cholinergic interneurons. (A) Representative images of M4 receptor (red) in ChAT-positive neurons (green) in DYT1 KI (DYT1) and wildtype (WT) mice, scale bar is 20 μm (B, C) quantification of respective fluorescence intensity in ChAT positive cholinergic interneurons (ChI); data shown as means + S.E.M., n = 6 each.

**Fig 6:**
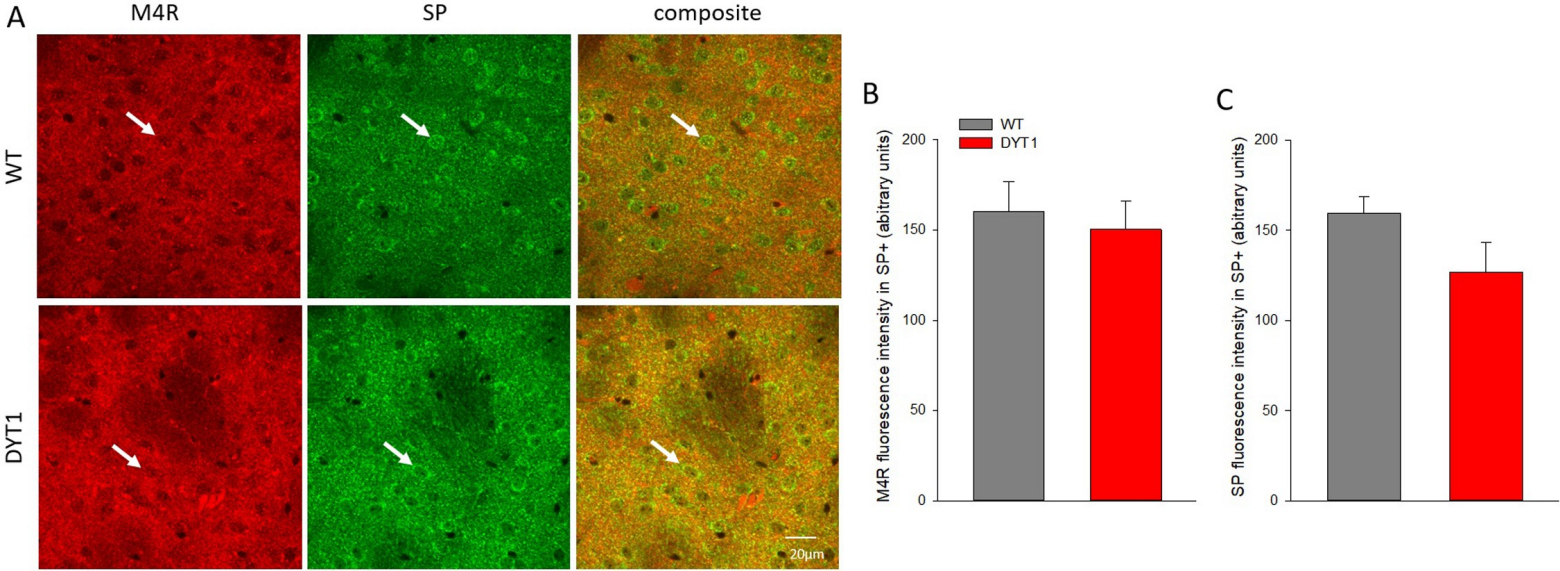
M4 receptor in substance P-positive neurons. (A) Representative images of M4 receptor (red) in substance P-positive neurons (SP+, green, white arrows) in DYT1 KI (DYT1) and wildtype (WT) mice, scale bar is 20 μm (B, C) quantification of respective fluorescence intensity in SP+ neurons; data shown as means + S.E.M., n = 6 each.

ChI are abundant in the lateral striatum, however, there was no difference in M4 receptor expression in somata of ChI (Fig 5) or SP-positive neurons (Fig 6) within the striatum. ChI could be readily identified because ChAT staining clearly discriminates somata (Fig 5A). Staining for SP identifies somata but also punctate structures of surrounding neuropil (Fig 6A), together concentrating in striosomes as shown previously with this antibody [6]. For specific quantification, M4 receptor fluorescence intensity was measured in neurons with strong and delineated SP-signal, which may have subsampled medium spiny neurons with high SP expression. Furthermore, as both ChAT and SP are cytoplasmic, using these markers for delineation will include mostly cytoplasmic M4 receptor signal. Nevertheless, the downregulation of M4R in the lateral striatum is unlikely to originate from the somata of ChI or SP-reactive SPN, but may instead indicate a slight reduction of M4 receptor on synaptic terminals. How and whether this contributes to the observed hypercholinergic phenotype of DYT1 KI mice would require further investigations. In addition, by determining the fluorescence intensity of ChAT, we could not detect changes of this acetylcholine synthetizing enzyme in ChI.

## Conclusions

In summary, our data support that changes in cholinergic transmission in DYT1 KI mice do not originate in or result from overt M1R or M4R expression levels, but from in part already previously described alterations in receptor function such as transmitter binding, plasma membrane localization or receptor signaling pathways. Cholinergic receptor expression is regulated in response to activation, but normal expression levels do not directly infer the presence of fully functional receptors. However, our findings have important implications for therapy: unspecific acetylcholine receptor antagonists are beneficial for some patients but side effects hamper therapeutic potential, therefore muscarinic receptor specific compounds are under development [20]. The present results support that the targeted receptors are available despite increase in cholinergic tone and previously reported alterations in dopamine receptors in DYT1 KI mice [26]. Additionally, to our knowledge, there are no data on abnormal muscarinic receptor expression in the caudate putamen of dystonia patients. Finally, our data also supports the recent shift in research focus towards nicotinergic receptors [24], which may in fact facilitate alterations found in dopamine signaling, and thus represent an interesting therapeutic target.

## Supporting information

S1 File Western blot images. (PDF)

## Abbreviations

ACh: Acetylcholine
ChI: cholinergic interneurons
SPN: striatal projection neurons
SP: substance P
