# Supplementary material for: Subtle changes in striatal muscarinic M1 and M4 receptor expression in the DYT1 knock-in mouse model of dystonia
Source: PLoS One. 2019 Dec 5;14(12):e0226080. doi: 10.1371/journal.pone.0226080 (PMC6894858; doi:10.1371/journal.pone.0226080)
Supplement: S1 File — (PDF) [file pone.0226080.s001.pdf]

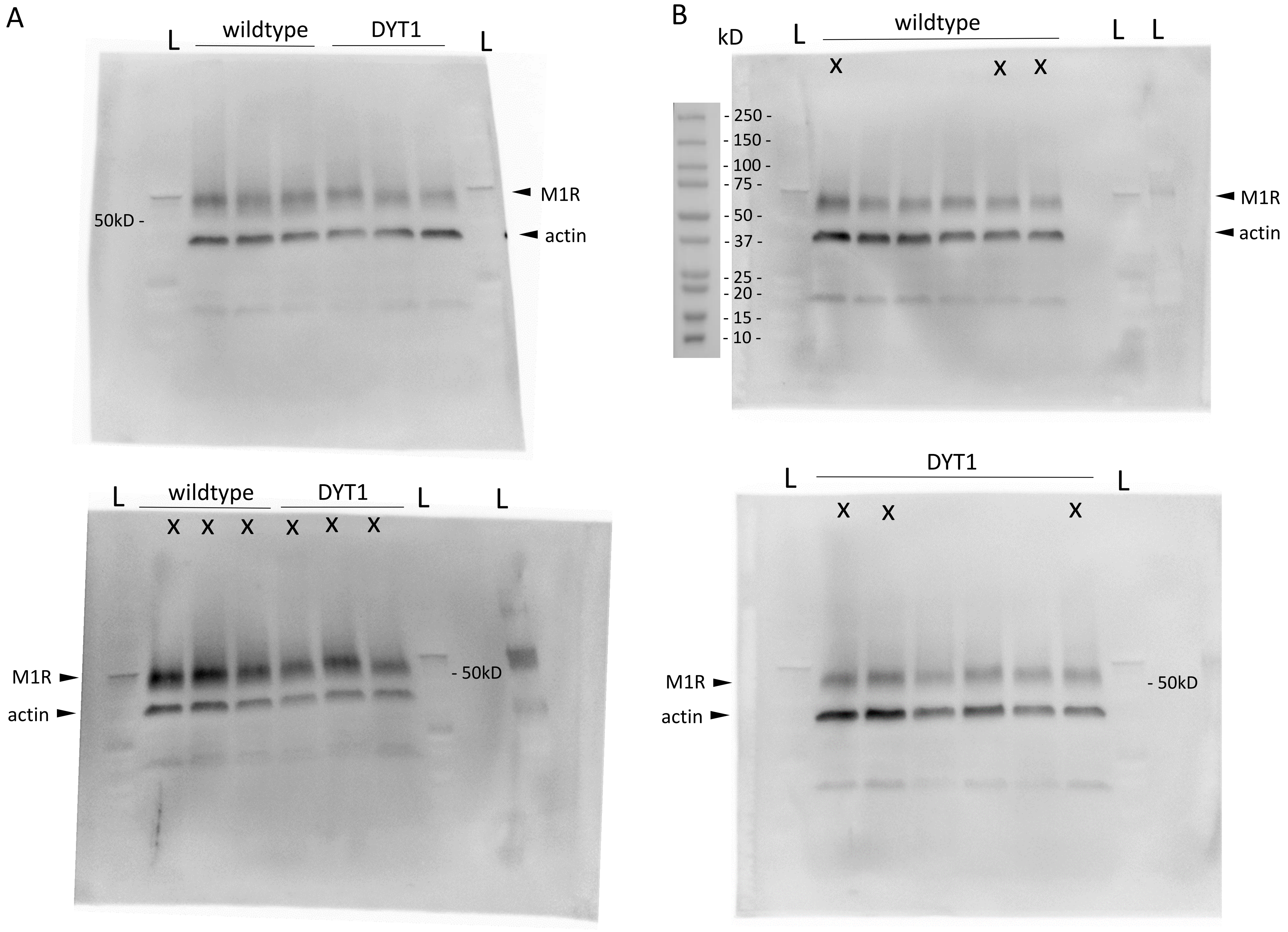

Suppl. Fig.: Western blot of M1 receptors in the (A) striatum and (B) motor cortex of DYT1 KI and wildtype mice. Blots with bands for M1 receptor ~ 51 kDa and actin ~ 40 kDa. No differences in expression between genotypes (mean + S.E.M.), M1 receptor expression was normalized to actin for quantification, n=6 each. blots in A or B were run together in one SDS-PAGE and transfer unit. Chemiluminescence visualized with FUSION advance imager with constant exposure times. Images were inverted for quantification, original full membrane images without modifications are shown.
